# Deadly Outbreaks: How Medical Detectives Save Lives Threatened by Killer Pandemics, Exotic Viruses, and Drug-Resistant Parasites

**DOI:** 10.3201/eid2002.131476

**Published:** 2014-02

**Authors:** J. Lyle Conrad

**Affiliations:** Centers for Disease Control and Prevention, Atlanta, Georgia, USA (reired)

**Keywords:** outbreaks, pandemics, viruses, drug-resistant parasites, malaria, West Nile virus, Legionnaires disease, medical detectives

As a reader of epidemiology literature and teaching materials since 1958, when a professor dropped the book Eleven Blue Men on my desk, I have tried to keep up with interesting outbreaks and studies. I bring to the attention of other epidemiologists the book, Deadly Outbreaks: How Medical Detectives Save Lives Threatened by Killer Pandemics, Exotic Viruses, and Drug-Resistant Parasites, by Alexandra M. Levitt, a recent collection of real and different outbreaks. It should be of interest not only to infectious diseases control specialists, but also to laboratory personnel and persons involved in studies that support epidemiologists in the field and in academic institutions.

Some of the outbreaks reported in this book, such as Legionnaires disease in Philadelphia in 1976, are relatively well known. Other outbreaks, such as West Nile fever in Queens, New York, in 1999 or drug-resistant malaria in Thailand in 1982, are not as well known to the general public.

The 7 sections in this book contain some of the latest laboratory advances and developments in subspecialties of virology, bacteriology, laboratory medicine, and environmental medicine. For example, who among us knew that channelopathy (progressive inflammatory neuropathy) would be a disease in humans working in pig slaughter houses?

This book is a reminder to all of us working in public health that our areas of interest are evolving in a variety of directions in the field and in research laboratories. This evolution is to be expected and is welcomed as we struggle to control new diseases that knock on our doors and open our eyes as we promote the interests of the rest of the world. It also reminds us that it takes all sorts of persons with a wide variety of backgrounds, interests, and abilities to respond to these new diseases and their associated problems and challenges. Our job is to respond quickly to limit these problems so that they have a minimal effect.

This book is a worthy addition to the libraries of health departments and schools of public health and medicine. It merits a place not too far away from where copies of Eleven Blue Men are shelved. Welcome to the modern epidemiology of the twenty-first century!

